# Gut microbiota as a regulator of vaccine efficacy: implications for personalized vaccination

**DOI:** 10.1080/19490976.2025.2563709

**Published:** 2025-10-06

**Authors:** Qi Lu, Yifei Feng, Haijian Wang, Kai Zhu, Lin Teng, Min Yue, Yan Li

**Affiliations:** a MOA Key Laboratory of Animal Virology & Zhejiang Provincial Engineering Research Center of Animal Biological Products, Zhejiang University College of Animal Sciences, Hangzhou, China; b Hainan Institute of Zhejiang University, Sanya, China; c Key Laboratory of Systems Health Science of Zhejiang Province, School of Life Science, Hangzhou Institute for Advanced Study, University of Chinese Academy of Sciences, Hangzhou, China

**Keywords:** Gut microbiota, vaccine efficacy, immunomodulation, dysbiosis, microbiota-immune axis, precision vaccinology

## Abstract

Vaccines are one of the most significant achievements in global health, as they have substantially reduced morbidity and mortality from infectious diseases. However, the vaccine efficacy varies markedly across different populations, particularly among infants, older adults, and people living in low- and middle-income countries. Host-intrinsic factors, such as sex, age, and genetic predisposition, contribute to these heterogeneities. However, accumulating data indicate that the gut microbiota also plays a pivotal role in modulating vaccine efficacy. This review summarizes current knowledge, demonstrating that vaccine efficacy is shaped not only by host biology but also by a dynamic, bidirectional interplay between the gut microbiota and immune system. We discuss how the microbiota influences vaccine outcomes through several mechanisms, including priming the innate immune response, regulating adaptive responses through metabolites, and facilitating antigen cross-reactivity. Furthermore, we examine the potential for microbiota-informed precision vaccinology, which integrates multiomics profiling and artificial intelligence to predict and improve vaccine performance. These advancements establish a framework for personalized vaccine development based on microbial ecology.

## 1 Introduction

Vaccination represents one of the most transformative achievements in global health. Initiatives like the Expanded Program on Immunization have prevented an estimated 154 million deaths over the past five decades.^1^ Nevertheless, significant heterogeneity in vaccine efficacy persists, particularly among high-risk populations such as older people, those with immunocompromising conditions, and those in low- and middle-income countries.^2^^,^^3^ Age-related immunosenescence, genetic polymorphisms, and comorbidities only partially explain this heterogeneity, suggesting that uncharacterized biological and environmental factors may further modulate immune responses to vaccines.^3^^,^^4^ Adjuvants are critical for enhancing vaccine efficacy, but ongoing concerns persist. These include safety issues, route-specific optimization (e.g., parenteral vs. mucosal delivery), and costs. Such concerns highlight the need for safer and cost-effective alternatives.^5^^,^^6^ Notably, emerging evidence suggests that the gut microbiota may act as a natural immunomodulator with adjuvant-like functions.^7^

The gut microbiota is a dynamic ecosystem that encodes approximately 150 times more genes than the human genome, serving as a critical regulator of systemic immunity.^8^^,^^9^ Beyond its metabolic functions, it modulates immune responses through diverse mechanisms: (i) innate immune priming through microbe-associated molecular patterns (e.g., flagellin-toll-like receptor 5 signaling)^10^; (ii) epigenetic reprogramming of B-cell metabolism by metabolites such as short-chain fatty acids (SCFAs)^11^; (iii) reprogramming of antigen-presenting cells^12^; and (iv) production of cross-reactive antigens that mimic vaccine targets.^13^ Critically, the composition and function of the microbiota are shaped by geographic, dietary, and socioeconomic factors. Consequently, distinct microbial ecosystems generate distinct immunological environments that directly influence vaccine efficacy. For example, urban recipients of the yellow fever 17D vaccine in Tanzania, whose gut microbiota is dominated by *Prevotella*, generate fewer neutralizing antibodies than do rural recipients.^14^ These findings position the microbiota as both a mediator and amplifier of host‒environment interactions, bridging gaps in our understanding of heterogeneous vaccine responses.

However, translating these insights into clinical applications remains challenging. While probiotics and postbiotics have the potential to restore gut microbiota homeostasis and enhance vaccine responses, their effectiveness is highly strain-specific and context-dependent.^15^^,^^16^ Recent advancements in high-throughput sequencing and artificial intelligence now enable the precise mapping of microbiota‒immune interactions, paving the way for the development of precision vaccination strategies tailored to individual microbial profiles.^17^^,^^18^

In this review, we summarize recent evidence linking microbiota composition and dynamics to heterogeneity in vaccine responses. We also explore the complex interactions between the gut microbiota and host immunity that influence vaccine efficacy. Furthermore, we introduce the concept of “microbiota adjuvant potential” as a means to predict vaccine outcomes and evaluate emerging strategies to integrate microbiota data into rational vaccine design.

## 2 Multifactorial determinants of vaccine efficacy

Vaccine efficacy varies significantly among individuals and populations due to a complex interplay of pathogen-related, vaccine-related, and host-specific factors.^19^ A comprehensive understanding of these determinants is critical for developing optimized immunization strategies.

### 2.1 Pathogen-related factors

Genetic and antigenic similarities between vaccine pathogens and circulating pathogens are crucial in influencing vaccine efficacy. The more similar the vaccine is to circulating pathogens, the more likely it is to have better efficacy. For example, the severe acute respiratory syndrome coronavirus 2 (SARS-CoV-2) Omicron variants exhibit increased immune evasion from neutralizing antibodies produced by natural infection and/or vaccination relative to earlier alpha or delta strains, primarily because of mutations in the spike protein that increase the affinity for the cellular receptor angiotensin-converting enzyme 2 (ACE2).^20^ In bacteria, *Streptococcus pneumoniae* undergoes serotype replacement following widespread vaccination with the heptavalent protein‒polysaccharide conjugate vaccine PCV7, which has driven increases in the nonvaccine serotype and vaccination failure.^21^ An analogous phenomenon is observed in *Bordetella pertussis*. The sustained use of acellular pertussis vaccines, which target pertussis toxin and adhesins such as pertactin, has selected for strains harboring *prn* gene deletions or mutations. Loss of pertactin expression circumvents vaccine-induced antibodies, thereby facilitating immune escape.^22^

### 2.2 Vaccine-related factors

Vaccine types, products, adjuvant formulations, doses, administration schedules, and pre-existing immunity jointly shape the magnitude and quality of immune responses to vaccines. mRNA vaccines to SARS-CoV-2, such as BNT162b2, elicit higher neutralizing antibody titers than inactivated vaccines, such as CoronaVac, whereas CoronaVac induces stronger CD4⁺ and CD8⁺ T-cell immunity to structural proteins.^23^ Extended intervals between priming and boosting improve efficacy against emerging SARS-CoV-2 variants. Hybrid immunity (natural infection followed by vaccination) enhances antibody production by activating memory B- and T-cell populations, underscoring the impact of prior immune exposure on vaccine efficacy.^24^^,^^25^

### 2.3 Host-specific characteristics

Age, sex, nutritional status, and gut microbiota substantially affect vaccine responsiveness. Immunosenescence refers to age-related declines in immune function, and inflammaging, characterized by chronic low-grade inflammation, can impair antibody and T-cell responses in older adults.^26^ Comorbidities, such as obesity, further exacerbate these impairments, as evidenced by reduced humoral responses to SARS-CoV-2 vaccines in individuals with metabolic disorders.^27^ Women generally mount stronger antibody responses to vaccines like those for influenza and hepatitis B. However, they tend to experience more frequent adverse reactions, potentially due to hormonal and genetic factors, including the expression of toll-like receptor 7 (TLR7) in B-cells.^28^ Yet some studies report higher SARS-CoV-2 vaccine efficacy in men, indicating unresolved complexities in sex-based immune regulation.^29^ Micronutrient deficiencies, iron and vitamin D in particular, impair lymphocyte proliferation and dendritic cell (DC) maturation, contributing to reduced vaccine efficacy in undernourished populations.^30^^,^ ^31^

The gut microbiota has emerged as a key regulator of systemic immunity, bridging host biology and environmental exposures. Dysbiosis, characterized by reduced microbial diversity or the dominance of proinflammatory taxa like *Enterobacteriaceae*, is correlated with impaired vaccine responses. For example, antibiotic-induced disruption of microbiota diversity and abundance has decreased neutralizing antibody titers after SARS-CoV-2 vaccination, confirming the role of the microbiota in fine-tuning immune outcomes.^32^ This interaction positions the gut microbiota as both a mediator and amplifier of traditional host factors, providing a foundation for mechanistic exploration in subsequent discussion.

## 3 From early colonization to dysbiosis: gut microbiota-immune interactions and their impact on vaccine efficacy

### 3.1 The colonization of intestinal microbiota in early life

The first 1000 d of life represent a critical window for gut microbiota colonization and immune system maturation.^33^ Early microbiota composition is shaped by delivery mode, maternal microbiota, antibiotic exposure, and feeding practices^34^. Neonates acquire initial colonizers from their environment, starting with facultative anaerobes. Over the first six months, these are followed by obligate anaerobes, such as *Bifidobacterium*, *Bacteroides*, and *Clostridium.*^34^ The delivery mode significantly affects colonization patterns: infants born vaginally inherit the maternal vaginal microbiota, in which *Lactobacillus*, *Prevotella*, and *Bifidobacterium* dominate.^35–38^ In contrast, infants born via cesarean section tend to have microbiota enriched in maternal skin and oral, such as *Staphylococcus* and *Corynebacterium*, together with opportunistic pathogens, including *Klebsiella* and *Enterococcus*. Even this early difference persists. At three months, cesarean section infants exhibit elevated *Firmicutes/Bacteroidetes* ratios, reduced *Bacteroidetes* and an increased abundance of opportunistic pathogens, such as *Clostridium sensu stricto 1, Enterococcus, Klebsiella, Clostridioides, and Veillonella*, which may increase long-term risks of immune dysregulation.^39^

The maturation of gut microbiota progresses through three phases: a breastfeeding-dominated phase (3−14 months), a transitional phase (15−30 months), and a stable phase (31−46 months).^40^ This progression is modulated by maternal factors (e.g., allergic status reducing the presence of *Bifidobacterium* in breast milk^41^), feeding practices (such as direct breastfeeding, which reduces pathogens in expressed milk), and environmental exposures (e.g., household hygiene and pet contacts^40^). Human breast milk oligosaccharides support the colonization of *Bifidobacterium* and *Bacteroides* while suppressing pathogens such as *Streptococcus* and *Enterococcus.*^42^ Breast milk-born cytokines, hormones, and bioactive peptides that shape infant gut microbiota by facilitating mucosal layer development.^43^ In contrast, formula feeding is associated with *Bifidobacterium* depletion and enrichment of antibiotic resistance genes.^44^^,^^45^ Exclusive breastfeeding initially leads to lower alpha-diversity in gut microbiota at 6 weeks; however, microbiota diversity rebounds and exceeds that of formula-fed infants by six months.^42^ After weaning, the microbiota maturation rapidly: *Firmicutes* expand^40^ and *Clostridium* and *Bacteroides* become dominant with body mass index rises.^46^

Environmental exposures modulate microbiota maturation. Exposure to household furry pets in prenatal or early life increases the abundance of *Ruminococcus* and *Oscillospira*, which correlates with increased risks of allergy and obesity in childhood.^47^^,^^48^ Antibiotics, especially amoxicillin and cefotaxime, markedly reduce beneficial bacteria (e.g., *Bifidobacterium*) while favoring enrichment of opportunistic pathogens (e.g., *Klebsiella*, *Enterococcus*).^49^ The dietary context is equally important. Western diets high in processed foods and low in fiber content disrupt the balance between *Bacteroidaceae* and *Prevotellaceae.*^45^ Traditional diets rich in unprocessed foods, fiber, and fermented foods that contain *Bifidobacterium*-based synbiotics enhance microbial diversity and maintain *Bifidobacterium* levels.^45^^,^^50^^,^^51^

### 3.2 Gut microbiota in shaping early-life immune maturation

The infant immune system develops through dynamic interactions with gut microbiota. Secretory immunoglobulin A (IgA) plays a key regulatory role by cross-reacting with commensal bacteria, such as *Bacteroidetes*. This interaction modulates the expression of polysaccharide utilization loci via Mucus-Associated Functional Factor (MAFF).^52^^,^^53^ Maternal transfer of RORγ^+^ regulatory T-cells and microbial metabolites stabilizes infant IgA levels across generations.^54^^,^^55^ In a prospective cohort, the prevaccination microbiome and metabolome of two-month-old infants predicted subsequent vaccine responses. High serum phenylpyruvate and increased abundance of lipid A biosynthetic genes are associated with stronger immunity.^56^ These data indicate that microbiota-targeted interventions in the first weeks of life could enhance vaccine efficacy.

Delivery mode influences these processes. In a birth cohort study of 101 infants, vaginal delivery was associated with significantly higher serum and mucosal IgG against a 10-valent pneumococcal conjugate vaccine (PCV-10) at 12 months and the meningococcal C vaccine at 18 months.^57^ These observations demonstrate a direct link between delivery route and vaccine-induced humoral immunity.

Gestational age is another critical determinant. Preterm infants exhibited distinct gut microbial development, characterized by elevated *α* microbiota diversity in the meconium but delayed gut microbial maturation. This delay involves persistent colonization by opportunistic pathogens such as *Klebsiella* and *Enterococcus*, alongside significantly delayed establishment of beneficial *Bifidobacterium* species. This dysbiotic colonization pattern is associated with heightened inflammatory tone and may underlie the attenuated vaccine responses observed in preterm populations.^58^

Bifidobacterial colonization in early life is particularly vital. Deficiency in *Bifidobacteria* is associated with systemic inflammation and immune dysregulation early in life.^59^ Conversely, supplementation with *B. longum* infantis EVC001 suppresses proinflammatory T helper 2 (Th2) and T helper 17 (Th17)-related cytokine expression while inducing immunoregulatory interferon beta (IFN-*β*) in infants.^59^ Furthermore, postweaning proliferation of *Lachnospiraceae* is correlated with a reduced risk of atopy.^60^

### 3.3 Gut microbiota and vaccine immunogenicity

The gut microbiota modulates vaccine immunogenicity through specific bacterial taxa such as *Bifidobacterium*, *Bacteroides*, *Clostridium* cluster XI, and *Proteobacteria*, as well as through metabolites like triterpenoids (Table 1). *Bifidobacterium* and *Bacteroides* enhance vaccine efficacy in infants.^61^ Colonization with *Bifidobacterium* infantis enhances BCG-specific T-cell responses in both infants and gnotobiotic mice,^62^ and higher *Bifidobacterium* abundance in early infancy is associated with stronger antibody responses to multiple pedialtric vaccines.^63^

**Table 1.** Impact of gut microbiota on vaccine immunogenicity.

Microbial species	Impact on vaccine efficacy	Refs.
*B. infantis*	Enhances BCG-specific T-cell responses in both infants and gnotobiotic mice	^62^
*Clostridium* cluster XI	Correlates with improved rotavirus vaccine seroconversion in Pakistani infants	^64^
*Proteobacteria*	Correlates with improved rotavirus vaccine seroconversion in Pakistani infants	^64^
*B. longum* BL999 + *L. rhamnosus* LPR	Enhances hepatitis B virus vaccine IgG responses in infants	^65^
*L. casei* GG	Enhances rotavirus vaccine-specific IgA and IgM seroconversion rates in infants	^66^
*E. coli* Nissle 1917	Enhances memory B-cell responses to rotavirus vaccine in antibiotic-treated piglets	^67^ ^,^ ^68^
*L. paracasei* 431	No change (influenza, adults)	^69^
*L. rhamnosus* GG (maternal)	Reduces pneumococcal and tetanus vaccine-specific IgG levels in infants	^70^
*L. plantarum* GUANKE	Enhances mucosal SARS-CoV-2 vaccine neutralizing antibodies by >8-fold in mice	^71^

In Pakistani infants, higher relative abundances of *Clostridium* cluster XI and *Proteobacteria*, including *Serratia*- and *Escherichia*-related species, correlate with improved rotavirus vaccine responses.^64^ Similarly, in Ghanaian infants, increased *Streptococcus bovis* abundance and reduced *Bacteroidetes* levels are linked to enhanced rotavirus vaccine immunogenicity, further highlighting the interplay between microbiota and vaccine outcome.^72^ Malawi and India infants have lower oral rotavirus vaccine shedding rates and seroconversion than UK infants. In these regions, greater microbial diversity was inversely correlated with oral rotavirus vaccine immunogenicity, suggesting that excessive early microbial exposure may potentially impair vaccine efficacy.^73^ However, the relationship between differences in global vaccination strategies and microbiota maturation stages remains unclear, indicating a need for further exploration of the mechanisms underlying microbiota‒immune interactions. Current evidence is insufficient to establish causality, emphasizing the need for additional research to understand these complex relationships fully.

### 3.4 Gut microbiota dysbiosis and vaccine failure

Vaccine failure refers to the inability to achieve protective immunity following vaccination. Vaccine-specific issues, such as improper storage, formulation defects, or mismatched serotypes, can contribute to vaccination failure.^74–76^ But host factors account for it, too. Immunosenescence, malnutrition, and immunosuppression hinder vaccine immunity. For example, immunosuppression reduces neutralizing antibody titers to tick-borne encephalitis virus vaccines.^77^

Emerging evidence underscores the gut microbiota as a key host factor influencing vaccine efficacy. Dysbiosis, defined as a disruption in microbial diversity and composition, has been linked to suboptimal immune priming. In neonates, exposure to antibiotic disrupts *Bifidobacterium* colonization, which correlates with diminished antibody responses to the 13-valent pneumococcal conjugate vaccine and the combined 6-in-1 Infanrix Hexa vaccine.^78^ Conversely, the abundance of *Clostridium perfringens* is inversely related to rotavirus vaccine responses, indicating strain-specific microbial associations.^79^ Probiotic interventions following antibiotic treatment show potential clinical benefits in restoring microbial balance and enhancing immunogenicity; however, their efficacy must be validated. These findings indicate the need for integrative vaccine optimization strategies that consider microbial ecology, host immunity, pathogen evolution, and vaccine design.^78^

### 3.5 Gut microbiota-mediated modulation of immune responses and vaccine efficacy

Evidence suggests that probiotics may modulate immune responses and enhance vaccine efficacy through interactions with gut microbiota.^4^ Causal evidence for the role of the gut microbiota in vaccine immunity comes from a landmark clinical study that depleted microbiota in humans using antibiotics.^80^ Antibiotic-mediated reduction in the gut bacterial load and diversity resulted in lower levels of rabies-specific IgG and neutralizing antibodies. Mechanistically, gut microbiota depletion skewed T-cell polarization (elevated Th1 frequencies and reduced circulating T follicular helper (cTfh) cells) and impaired germinal-center B-cell maturation and somatic hypermutation.^80^ These studies establish causality, but strain specificity and host context should also be considered.

*Bifidobacterium* and *Lactobacillus* strains function as live adjuvants, though their efficacy is strictly strain- and context-dependent. In infants receiving oral rotavirus vaccine, supplementation with *Bifidobacterium longum* BL999 and *Lactobacillus rhamnosus* LPR enhanced antibody titers against hepatitis B virus surface protein.^65^ Similarly, *Lactobacillus casei* GG significantly increased the rates of rotavirus-specific immunoglobulin M (IgM) and IgA seroconversion in infants.^66^ Animal models further support these strain-specific effects: *Escherichia coli* Nissle 1917 enhances memory B-cell activation and reduces rotavirus shedding in antibiotic-treated piglets,^67^^,^^68^ while *Lactobacillus plantarum* GUANKE boosted SARS-CoV-2 neutralizing antibodies by >8-fold in mucosal compartments in post-vaccinated mice.^71^ Conversely, *Lactobacillus paracasei* 431 failed to enhance influenza virus-specific IgG or IgA levels in adults,^69^ and maternal *Lactobacillus rhamnosus* GG supplementation reduced pneumococcal and tetanus-specific IgG in infants, possibly due to excessive regulatory T-cell induction.^70^ These contradictions underscore a strain-dependent adjuvant-like effect that relies on genomic repertoires, secreted metabolites, and immunomodulatory molecules,^81–83^ thereby dictating interactions with host pattern recognition receptors (PRRs) like TLRs and NOD-like receptors on innate immune cells.^10^^,^^84^

Critically, microbial immunostimulatory capacity may reside not in the viability or taxonomic identity of the bacterium itself, but rather in the expression of specific microbial-associated molecular patterns and their ability to engage defined innate immune pathways.^85^ Heat-killed *E. coli* and TLR2 ligands Pam3CSK4 robustly upregulated CD11b expression on IgA⁺ B-cells in Peyer's patches, facilitating B-cell entry into the germinal center and mucosal antigen-specific IgA production via NOD2 signaling pathway, but live *Bifidobacterium* species failed to do so.^85^ Defined ligands may therefore outperform traditional probiotics as predictable, safe mucosal adjuvants.

The evidence highlights that probiotic effects are species- and strain-specific, and are further modulated by host age, existing microbiota, genetics, and timing of administration. Advancing from generic probiotics to precise interventions—targeting defined microbial functions, metabolites or personalized restoration—will be essential for the rational enhancement of vaccine efficacy.

## 4 Vaccine efficacy heterogeneity: interplay between gut microbiota and intrinsic and extrinsic factors

**Figure 1.**
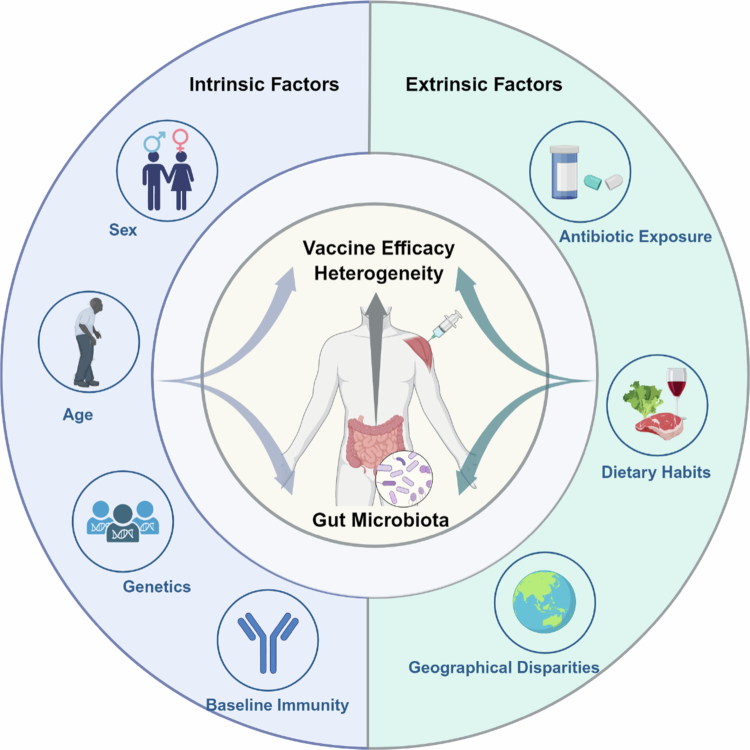
Interactions between intrinsic and extrinsic factors with the gut microbiota shape heterogeneity in vaccine efficacy.

Host factors, vaccine-related factors, and the gut microbiota modulate vaccine efficacy.^4^ Increasing evidence indicates that intrinsic host characteristics and extrinsic environmental factors interact with the gut microbiota, driving significant heterogeneity in immune responses to vaccines (Figure 1). This section examines these interactions through the dual lenses of intrinsic and extrinsic factors.

### 4.1 Intrinsic factors influencing vaccine efficacy via gut microbiota

Host factors, including sex, age, genetics, and pre-existing immunity, are established determinants of vaccine-induced immune responses.^19^^,^^86^^,^^87^ Emerging evidence indicates that these factors interact dynamically with the gut microbiota composition and function to modulate vaccine efficacy^88^ (Table 2).

**Table 2.** Intrinsic factors influencing vaccine efficacy via gut microbiota.

Intrinsic factor	Impact on gut microbiota	Impact on vaccine efficacy	Refs.
Sex differences	Sex-specific composition; hormone-mediated shifts (e.g., androgen depletion → female-like profiles); antibiotic treatment attenuates sex-associated microbial-immune disparities	XX mice: elevated IgM-secreting B-cells/plasma cells vs. XY; microbiota depletion abrogates enhanced humoral responses in XX mice; sex-specific shifts post-SARS-CoV-2 vaccine (males: *Succinivibronaceae*; females: *Desulfovibrionaceae*)	^89^
Aging effects	Reduced beneficial taxa (*Bifidobacterium*, *Clostridiales*); increased pro-inflammatory *Proteobacteria* (*Enterobacteriaceae*); diminished diversity	Reduced vaccine efficacy; lower neutralizing antibody titers in older adults (e.g., BBIBP-CorV, mRNA-1273)	^90^
Genetic polymorphisms	TLR5 loss-of-function impairs flagellin sensing; Ripk2 deficiency disrupts bacterial peptidoglycan-mediated signaling	Reduced antibody responses to non-adjuvanted vaccines (e.g., influenza in TLR5-deficient mice); blunted mucosal immunity/oral vaccine efficacy (Ripk2 deficiency)	^10^ ^,^ ^84^
Pre-existing immunity	Obesity-associated dysbiosis; HIV infection enriches *Enterobacteriaceae*; pro-inflammatory taxa disrupt balance; commensals (*Bifidobacterium*) support homeostasis	Obesity attenuates SARS-CoV-2 vaccine humoral responses; HIV compromises vaccine immunogenicity; hybrid immunity (infection + vaccination) enhances antibody production	^27^ ^,^ ^91^

#### 4.1.1 Sex-specific interactions between gut microbiota and immunity

Sex differences significantly influence the gut microbiota composition and immune function.^86^ contributing to immune sex bias in autoimmune diseases and the heterogeneous immune response to vaccines.^86^^,^^92^

In nonobese diabetic mice, females exhibit a higher incidence of type 1 diabetes than males; however, this disparity disappears under germ-free conditions, suggesting that the microbiota plays a role in sex-based divergence.^92^ Steroid hormones further mediate this relationship: castrated male mice develop female-like gut microbial profiles, while androgen depletion correlates with an increased incidence of autoimmune diseases in males.^92^ Antibiotic treatment in primary biliary cholangitis models attenuates sex differences in lymphocytic infiltration and inflammatory cytokine production, confirming the role of the microbiota in sex-specific immune regulation.^93^

The microbiota-sex-immune axis also affects vaccination outcomes. Amato-Menker et al. demonstrated that the sex chromosome complement directly influences the gut microbiota-mediated immune response to vaccination with heat-killed *Streptococcus pneumoniae.*^89^ Mice with XX chromosomes exhibited elevated IgM-secreting B-cells and plasma cells compared to XY mice, with upregulated expression of the X-linked epigenetic regulator *Kdm6a*. Microbiota depletion abrogated these enhanced humoral responses in XX mice, while short-chain fatty acid-producing bacteria restored immunogenicity.^89^ Consistent with this, a recombinant yeast vaccine expressing the SARS-CoV-2 spike protein altered microbiota composition in a sex-specific manner: males enriched *Succinivibronaceae*, *Atopobiaceae,* and *Akkermansiacea*e, whereas females enriched *Desulfovibrionaceae.*^94^ Such bioengineering strategies may help optimize vaccine design by taking into account the sex-specific microbial and immunological landscapes.^86^

#### 4.1.2 Age-related gut microbiota changes and vaccine efficacy

Aging alters gut microbiota and immune function, reducing vaccine efficacy.^61^^,^^87^ Aging-associated microbial shifts include a reduction in beneficial bacteria like *Bifidobacterium* and *Clostridiales*, an increase in pro-inflammatory *Proteobacteria* (e.g., *Enterobacteriaceae*) and diminished diversity.^90^ This dysbiosis drives immunosenescence, characterized by chronic low-grade inflammation (termed “inflammaging”) and impaired adaptive immunity.^90^ Phase 1/2 trial of BBIBP-CorV showed significantly lower neutralizing antibody titers in adults aged 60 y or older compared to younger cohorts across all doses.^95^ Similarly, Moderna's mRNA-1273 elicited lower neutralizing antibody titers assessed by the PRNT80 assay in participants ≥71 y than in the 56~70-y age group.^96^ These findings highlight how aging-associated gut microbiota dysbiosis and immunosenescence compromise vaccine outcomes. Probiotic interventions (e.g., *Bifidobacterium* supplementation) or dietary modifications may offer a promising strategy to increase vaccine efficacy in aging populations.^90^

#### 4.1.3 Host genetic polymorphisms and gut microbiota in vaccine efficacy

Host genetic polymorphisms in immune regulatory pathways (e.g., Toll-like receptors, NOD-like receptors, and cytokine genes) critically shape the gut microbiota composition and modulate vaccine efficacy. For instance, TLR5 loss-of-function variants impair the sensing of microbiota-derived flagellin and reduce antibody responses to nonadjuvanted vaccines, such as influenza, in mice.^10^ Deficiency in Ripk2, the adaptor required for Nod1/Nod2 signaling, blunts mucosal immunity and reduces oral vaccine efficacy due to disrupting bacterial peptidoglycan-mediated signaling transduction.^84^ Thus, host genetics and the microbiota engage in reciprocal tuning of immune thresholds.

#### 4.1.4 Pre-existing immune status and gut microbiota interactions in vaccine efficacy

Pre-existing immune status, shaped by prior infections, chronic inflammatory conditions, and immunological memory, modulates vaccine efficacy through interactions with the gut microbiota.^4^ Systemic inflammation resulting from comorbidities, such as obesity or autoimmune disorders, alters microbial composition, and metabolite profiles, disrupting immune cell function. For instance, obesity-associated microbiota profiles may propagate low-grade inflammation and attenuate humoral responses to SARS-CoV-2 vaccine.^27^ Similarly, chronic human immunodeficiency virus (HIV) infection depletes CD4⁺ T-cells, enriches *Enterobacteriaceae* to promote dysbiosis, compromises the gut barrier, and collectively dampens vaccine immunogenicity.^91^ Conversely, immunological memory shaped by prior pathogen exposure further modulates vaccine outcomes.^25^ Hybrid immunity (natural infection followed by vaccination) activates memory B-cells and enhances antibody production.^25^^,^^97^ Microbiota-derived signals, such as short-chain fatty acids, may amplify these processes,^11^ although causal evidence remains limited. Critically, the gut microbiota regulates baseline inflammation and immune memory equilibrium. Proinflammatory taxa (e.g., *Enterobacteriaceae*) can shift this balance toward immune tolerance or hyperactivation. In contrast, commensal bacteria (e.g., *Bifidobacterium*) help reduce excessive inflammatory responses and maintain immune homeostasis.^59^

### 4.2 Extrinsic factors influencing vaccine efficacy via gut microbiota

Extrinsic factors, including antibiotic exposure, dietary habits, and geographical disparities, modulate the gut microbiota composition and function, thereby influencing immune responses and vaccine efficacy^73^^,^ ^98^^,^^99^ (Table 3).

**Table 3.** Extrinsic factors influencing vaccine efficacy via gut microbiota.

Extrinsic factors	Specific factors	Impact on gut microbiota	Impact on vaccine efficacy	Refs.
Antibiotic exposure	Broad-spectrum Antibiotics	10,000-fold reduced microbiota load; depleted *Lachnospiraceae*; lower secondary bile acids	Reduced H1N1-specific IgG1/IgA titers; weakened immune response	^98^
	Narrow-spectrum Antibiotics	Enriched *Prevotellaceae*	Improved early rotavirus vaccine immunogenicity (day 7)	^100^
Dietary habits	Fiber-rich, minimally processed diets	Diverse, stable microbiota; metabolite-mediated epigenetic regulation	Supported immune modulation; potential efficacy boost	^101^
	Protein-calorie malnutrition	Lower *Firmicutes/Bacteroidetes* ratio; increased *Proteus*; reduced *Turicibacter*; gut barrier dysfunction	Reduced oral vaccine seroconversion; poor rotavirus vaccine response in children	^102^
	Vitamin A deficiency	Increased *Bacteroides vulgatus*; disrupted bile acid metabolism	Reduced mucosal immunity; weakened oral vaccine response	^103^
	Zinc deficiency	Microbial zinc competition; potential dysbiosis	Reduced Th1/IgG responses; lower Hepatitis B vaccine efficacy	^104^ ^,^ ^105^
Geographical disparities	Differences in sanitation, pathogen burden, and antibiotic use	High diversity in low-income regions (India/Malawi infants; Tanzania rural: *Prevotella*) Low diversity in urban areas (*Bacteroides* enrichment)	Negative correlation between diversity and ORV immunogenicity (India/Malawi); higher yellow fever Ab titers in rural Tanzania	^14^ ^,^ ^73^

#### 4.2.1 Antibiotic modulation of gut microbiota and its impact on vaccine efficacy

Antibiotic interventions influence vaccine efficacy in a context-dependent and spectrum-specific manner by altering the gut microbiota–immune interactions.^98^ The use of broad-spectrum antibiotics, including vancomycin, neomycin, and metronidazole, both before and after seasonal influenza vaccination, reduced the gut microbiota load by 10,000-fold and depleted immunomodulatory *Lachnospiraceae* for over six months. This dysbiosis resulted in a significant reduction of H1N1-specific neutralizing and IgG1/IgA titers in seronegative individuals. This deficit was linked to diminished secondary bile acids, particularly lithocholic acid, which restrains NLRP3 inflammasome activation and supports DC homeostasis. Antibiotic treatment also enhances proinflammatory AP-1/NR4A transcriptional signatures resembling immunosenescence.^98^

Conversely, narrow-spectrum vancomycin improved early immunogenicity (day 7) even in individuals with high existing IgA levels to rotavirus vaccines.^100^*Prevotellaceae* enrichment emerged as a biomarker distinguishing immunogenicity boosters from viral shedders, suggesting that microbiota-dependent antigen processing.^100^ Thus, broad-spectrum antibiotics generally impair vaccination by disrupting microbial–metabolic–immune networks, whereas targeted, narrow-spectrum depletion may transiently prime immunity. Replenishing keystone taxa or restoring bile-acid profiles may mitigate antibiotic-associated deficits.

#### 4.2.2 Dietary habits, gut microbiota, and their influence on vaccine efficacy

Diet critically determines gut microbiota diversity and function, thereby modulating vaccine efficacy.^99^ Fiber-rich, minimally processed diets foster stable and diverse microbial communities that produce metabolites capable of epigenetically regulating host metabolism.^106^ Conversely, protein-calorie malnutrition induces intestinal dysbiosis, epithelial barrier dysfunction, metabolic perturbations, and environmental enteropathy, thereby reducing seroconversion rates for oral vaccines and is associated with poor rotavirus vaccine efficacy in children.^101^^,^^107^^,^^108^ In pig models, protein deficiency reduced protection against human rotavirus diarrhea, suppressed innate and adaptive immune responses, and increased viral shedding; these changes coincided lower *Firmicutes/Bacteroidetes* ratio, upregulation of pro-inflammatory *Proteus*, and loss of immunocompetence-associated *Turicibacter.*^102^ These microbial shifts likely impair vaccine efficacy through dysregulated cytokine signaling and compromised antigen presentation.

Micronutrient deficiencies exacerbate the problem. Vitamin A deficiency, which is common in low- and middle-income countries, impairs mucosal immunity and immune responses to oral vaccines. Germ-free pig models have revealed diminished rotavirus vaccine efficacy in the presence of vitamin A deficiency.^109^^,^^110^ Vitamin A deficiency also increases the abundance of *Bacteroides vulgatus*. It perturbs bile acid metabolism, further reciprocally impairing vitamin A absorption.^103^ Similarly, zinc deficiency impairs Th1 and IgG responses to vaccines. Zinc-deficient mice showed reduced hepatitis B vaccine efficacy, potentially due to microbial competition for zinc, which limits the benefits of oral zinc supplementation.^104^^,^^105^

#### 4.2.3 Geographical disparities in gut microbiota and vaccine efficacy

Regional differences in sanitation, pathogen burden, and antibiotic use generate geographically distinct microbiota profiles that correlate with vaccine performance. For example, infants in the UK exhibit significantly higher rates of vaccine shedding and seroconversion than those in India and Malawi.^73^ This disparity is associated with gut microbiota diversity: infants in Malawi and India experience greater early-life microbial exposure, reflected in greater microbiota diversity and enrichment.^73^ Similar geographical differences influence responses to the yellow fever vaccine. In Tanzania, rural populations harbor distinct gut microbiota profiles characterized by higher diversity and enrichment of taxa such as *Prevotella* and *Succinivibrio*, which may correlate with a fiber-rich diet and local fermented foods.^14^ Concurrently, rural residents, who exhibit higher microbiota diversity and a higher abundance of *Bacteroides*, have higher neutralizing antibody titers to the yellow fever vaccine at 4 weeks postvaccination.^14^ These studies suggest that environmental factors sculpt microbiota–immune interactions, creating a dual challenge in low- and middle-income countries where high infectious pressure and suboptimal vaccine efficacy coexist.

## 5 Mechanistic insights into gut microbiota modulation of vaccine efficacy

The gut microbiota profoundly influences vaccine efficacy through interconnected mechanisms, including innate immune activation, metabolite-mediated signaling, antigen-presenting cell reprogramming, and antigen cross-reactivity.^4^ In this section, we integrate clinical and preclinical evidence to elucidate how these pathways synergistically influence immune responses, highlighting their translational potential for optimizing vaccine strategies.

### 5.1 Innate immune sensing of microbial signals

**Figure 2.**
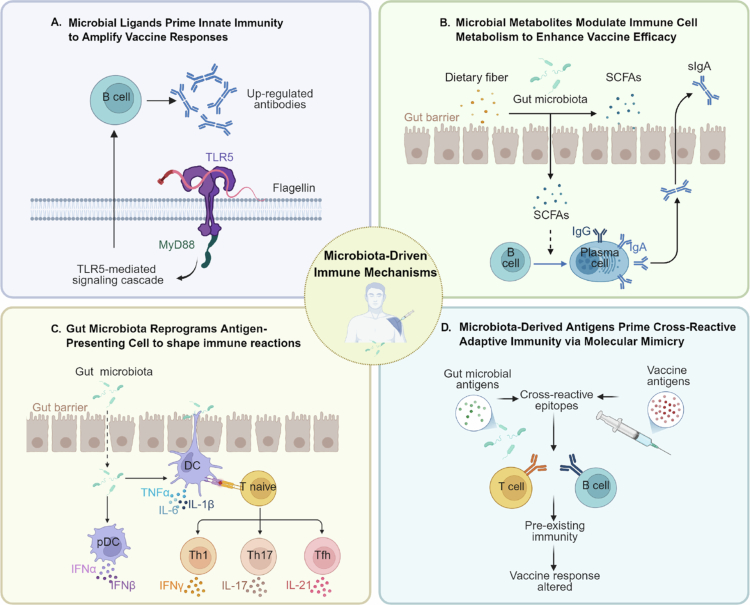
Mechanisms linking gut microbiota to vaccine immunogenicity and efficacy.

The gut microbiota serves as a reservoir of natural adjuvants that prime vaccine-induced immunity via pattern recognition receptors (Figure 2A). Flagellin from commensal *Proteobacteria* (e.g., *E. coli*) stimulates TLR5 on activated B-cells and macrophages, enhancing antibody class switching and elevating neutralizing titers against seasonal influenza vaccines in mice^10^. In humans, TLR5 expression on peripheral blood mononuclear cells correlates with higher antibody titers to hemagglutination after vaccination.^111^ Antibiotic depletion of flagellin-producing bacteria reduces influenza vaccine-induced IgG1 responses, and TLR5-deficient mice partly recapitulate this defect.^10^

NOD2 recognizes peptidoglycan and is required for mucosal adjuvanticity. Cholera toxin enhances NOD2-cAMP signaling in CD11c⁺ cells, driving follicular helper T-cell differentiation and germinal center plasma cell formation.^84^ Paradoxically, dysbiosis-induced overgrowth of *Bacteroides*, which is common in low-income populations, may reduce innate immune activation. This mechanism may explain the diminished IgA titer to oral rotavirus vaccines in low- and middle-income countries, where *Enterobacteria/Bacteroides* ratios are high.^72^

### 5.2 Gut microbiota-derived metabolites regulate vaccine efficacy

Microbial metabolites modulate immune cell activity through epigenetic and metabolic reprogramming, critically shaping vaccine responsiveness (Figure 2B). Short-chain fatty acids SCFA such as butyrate, enhance oxidative phosphorylation in B-cells and promote plasma cell differentiation, thereby boosting antibody production against *Citrobacter rodentium.*^11^ High-fiber diets that increase SCFA levels are correlated with stronger immune responses to SARS-CoV-2 vaccines.^112^ Perturbation of secondary bile acids, particularly lithocholic acid, by broad-spectrum antibiotics elevates NLPR3 inflammasome signaling, reducing vaccine-induced IgG1 antibodies specific to the H1N1 A/California strain in immunologically naïve adults.^98^*Bifidobacterium* infantis-derived indole-3-lactic acid enhances mucosal immunity by suppressing Th2 and Th17 inflammation.^59^ Feng et al. linked the dysbiosis-induced loss of circulating secondary bile acids (e.g., deoxycholic acid and lithocholic acid) and increases in fatty acylcarnitines to Th17/Treg imbalance, B-cells dysfunction and reduced rabies-specific humoral responses.^80^

### 5.3 Reprogramming of antigen-presenting cell via gut microbiota

The gut microbiota regulates vaccine-induced immunity by modulating antigen-presenting cell function through direct microbial signaling and metabolite regulation (Figure 2C). Dendritic cells (DCs), including conventional DCs (cDCs) and plasmacytoid DCs (pDCs), are primary targets of microbiota-driven reprogramming. Oral administration of *Bacteroides dorei* up-regulates CD80 and CD86 on conventional DCs and increases IL-12 secretion, resulting in higher spike-specific IgG and neutralizing antibodies to adenovirus-based SARS-CoV-2 vaccines in mice.^113^ Antibiotic-induced depletion of gut bacteria reduces the production of secondary bile acids (e.g., deoxycholic acid), which promotes DC activation and the elevation of AP-1/NR4A-driven inflammatory cytokines IL-6 and IL-8, thereby impairing vaccine responses.^98^ Conversely, plasmacytoid DCs constitutively produce type I interferon in response to microbial signals, which metabolically primes conventional DCs for efficient T-cell priming.^12^ Germ-free mice exhibit defective T-cell responses to influenza virus infection, confirming the importance of the microbiota in determining vaccine responsiveness.^114^

### 5.4 Gut microbiota-mediated antigen cross-reactivity and immune memory

The gut microbiota shapes pre-existing immunity through molecular mimicry and the modulation lymphocyte repertoire selection (Figure 2D). CD4⁺ memory T-cells specific for pathogen epitopes are detectable in individuals without prior infection, implying cross-reactivity with commensal microbial antigens.^115^ Bioinformatic analyses of T-cell receptor repertoires reveal shared epitopes between the human proteome, gut microbiota, and pathogenic bacteria.^116^*Enterococcus hirae* encodes a bacteriophage epitope that induces CD8⁺ T-cells cross-reactive with tumor antigens and enhances antitumor immunity in mice.^117^ Similarly, human and murine gut commensal-derived heat-shock proteins 60 and 70 from *E. coli* share sequence homology with the SARS-CoV-2 spike protein’s S2 hinge region, especially at P144. Monoclonal antibodies raised against these microbial proteins neutralize SARS-CoV-2 and enhance antibody responses to SARS-CoV-2 DNA vaccination.^13^ Such cross-reactivity may prime or divert vaccine antigen recognition.

Sequential microbial exposures diversify antibody repertoires but may also impair pre-existing immune responses. A high pathogen burden in low-resource settings reduces the efficacy of oral rotavirus vaccines.^73^^,^^118^ Pre-existing memory B-cells cross-reactive to commensal microbial epitopes can also direct HIV-1 Env gp41 vaccination toward a nonneutralizing gp41 antibody response, suggesting that modulating the gut microbiota composition may promote more protective anti-HIV-1 Env antibody induction.^119^ These dual roles underscore the need for precise microbiota modulation to avoid immune interference.

## 6 Microbiota-guided vaccination: from empirical adjuvants to precision strategies

Significant heterogeneity in vaccine responses, which is partially mediated by the gut microbiota, has driven growing interest in microbiota-based interventions to enhance vaccination outcomes. Integrating traditional methodologies with cutting-edge technologies, such as multiomics profiling and artificial intelligence (AI), enables the development of personalized vaccination frameworks tailored to an individual’s microbial and metabolic signatures. This section reviews conventional approaches and technological innovations and examines precision vaccinology strategies that support such precision approaches.

### 6.1 Conventional approaches and their limitations

Traditional strategies to enhance vaccine efficacy through gut microbiota modulation have relied primarily on the use of probiotics and postbiotics. Certain *Bifidobacterium* and *Lactobacillus* strains activate T-cells, thereby enhancing antibody production in response to SARS-CoV-2 vaccines.^120^ Postbiotics, which include heat-killed bacteria, microbial components, and metabolites, have emerged as promising alternatives to probiotics.^7^ The utilization of postbiotics eliminates the risks of antibiotic resistance gene transfer, withstands environmental stressors more robustly, and requires no viability maintenance while retaining immunomodulatory functions.^15^ For example, heat-killed *L. paracasei* MCC1849 boosted antibody titers to influenza vaccines in elderly populations aged 85 y or older.^121^ Despite these advances, significant limitations persist. Probiotic effects are highly strain-specific and face safety concerns and regulatory hurdles, including FDA approval requirements. Postbiotics, although generally safer, may trigger unintended immunogenic reactions through systemic absorption.^7^

Broader implementation of microbiota-guided vaccine strategies faces additional challenges. (i) Ethical considerations: Interventions in vulnerable populations (infants, elderly, immunocompromised) risk unforeseen immune activation, dysbiosis, and antimicrobial resistance spread; (ii) Technical barriers: standardized protocols for microbiota assessment, taxonomic annotation, and functional validation are lacking, impeding cross-study comparisons; (iii) Economic constraints: high-throughput sequencing, longitudinal immune monitoring, and GMP-grade therapeutic development are costly and aggravate financial burdens for low-resource settings; (iv) Generalizability limitations: Most evidence derives from high-income populations, leaving low- and middle-income populations under represented. While probiotics and postbiotics show promise, their integration into precision vaccinology requires overcoming these multifaceted hurdles.

### 6.2 Precision vaccinology: predictive monitoring and personalized interventions

Advancing precision vaccination strategies involves two complementary approaches: targeted gut–immune axis modulation and advanced technology-driven response prediction (Table 4).

**Table 4.** Strategies targeting the gut microbiota to enhance vaccine efficacy.

Strategy	Approach	Key evidence	Refs.
Probiotics	Certain *Bifidobacterium* and *Lactobacillus* strains	Activated T-cells and augmented antibody production to SARS-CoV-2 vaccines	^120^
Postbiotics	Heat-killed *Lactobacillus paracasei* MCC1849 administration	Improved influenza vaccine antibody titers in elderly populations ( ≥ 85 y)	^121^
Engineered probiotics	Recombinant yeast expressing SARS-CoV-2 spike protein	Sex-specific microbiota modulation: males enriched *Succinivibrionaceae*, *Atopobiaceae*, *Akkermansiaceae*; females enriched *Desulfovibrionaceae*	^94^
Defined microbial ligands	Heat-killed *E. coli*/TLR2 ligands (Pam3CSK4) administration	Upregulated CD11b on IgA⁺ B-cells in Peyer's patches, enhancing germinal center entry and mucosal IgA production	^85^
Predictive biomarkers	Machine learning integrating multi-omics	*Streptococcaceae/Enterobacteriaceae* enrichment + secondary bile acid depletion correlated with reduced rabies vaccine IgG/neutralizing antibodies; models stratify low-responders	^80^

#### 6.2.1 Technological innovations for translating microbiota insights into clinical practice

Next-generation sequencing (metagenomics, metabolomics) and multiparametric flow cytometry enable high-resolution characterization of microbiota–immune dynamics. Machine learning frameworks integrating multiomics datasets identify gut microbial biomarkers. For example, a reduced abundance of *Bacteroides* and *Streptococcus* correlates with elevated proinflammatory cytokines IL-6 and TNF-*α* and predicts disease severity.^122^ These models could stratify individuals into high- or low-response cohorts for prevaccination interventions. A landmark study integrated metagenomics, transcriptomics, and metabolomics with machine learning to construct a multiscale and multiresponse network.^80^ This network revealed that antibiotic treatment led to the enrichment of *Streptococcaceae* and *Enterobacteriaceae*, with reduced secondary bile acids correlated with Th1-biased CD4⁺ T-cell polarization and impaired rabies-specific antibodies.^80^ These findings validate microbiota modulation as a therapeutic strategy and highlight personalized approaches, such as secondary bile acid restoration or defined probiotic usage, to recalibrate microbial–immune crosstalk.

#### 6.2.2 Vaccine-specific microbiota requirements

Multiomics analyses have identified microbial communities that respond to vaccines, characterized by key taxa, shared metabolites, and high *α*-diversity.^123^ Different classes of vaccines interact with these communities in distinct ways. (i) Live-attenuated oral vaccines, such as the oral poliovirus vaccine, require a balanced gut microbiota. When antibiotics reduce microbial diversity, they lead to a decrease in poliovirus-specific IgA, particularly when *Clostridia* are abundant and *Bifidobacterium* is scarce.^124^ (ii) Inactivated vaccines, such as seasonal influenza vaccines, rely on secondary bile acids derived from the microbiota. Antibiotic-induced depletion of gut bacteria can lower these metabolites ~1000-fold and impair hemagglutinin-specific IgG1/IgA titers and neutralizing antibodies.^98^ (iii) Subunit vaccines, such as the hepatitis B virus vaccine, benefit from probiotic adjuvanticity; perinatal supplementation with blends of *Lactobacillus/Bifidobacterium* has been associated with increased anti-HBsAg titers, although host factors can influence the extent of this response.^65^ (iv) Conjugate bacterial vaccines, such as pneumococcal conjugate vaccines and *Haemophilus influenzae* type b vaccines, prove less effective after antibiotic exposure in infants. This results in lower antibody titers linked to a decrease in *Bifidobacterium* levels and *α*-diversity.^123^ These findings reveal the specific dependencies of various vaccine types on microbiota composition and support that timing probiotic or prebiotic interventions around vaccination can help personalize immunogenicity improvements.

#### 6.2.3 Interplay of vaccination routes and microbiota

The route of administration significantly influences how the microbiota affects the immune response. Oral vaccines, such as those for cholera and rotavirus, directly engage Peyer’s patches. In this process, resident commensal bacteria or bacterial antigens stimulate the expression of integrin CD11b on pregerminal center IgA^+^ B-cells, which drives mucosal immune responses.^85^ In contrast, parenteral vaccines, such as the rabies vaccine, prime systemically. The effectiveness of these vaccines may rely on microbial metabolites, such as secondary bile acids.^80^ Vaccines administered at mucosal sites, such as intranasal influenza vaccines, activate local inductive tissues but also depend on distal gut–lung axis signals through Toll-like receptor pathways. This signaling pathway supports the production of respiratory IgA production and T-cell responses in the lungs.^125^ Thus, microbiota-targeted interventions should be tailored to match both the existing microbial community and the chosen route of vaccine delivery.

#### 6.2.4 Persisting challenges and future directions

Key challenges persist in the field. These include the lack of standardized protocols for probiotic strain, dose, timing, as well as information on the existing microbiota and host genetics.^126^ AI-driven predictive platforms must also account for population diversity, including variations in ethnicity, age, and comorbidities, to ensure generalizability. For example, fecal metabolomics has revealed amino acid-related pathways that link microbial metabolism to inflammation. However, these findings require validation across diverse cohorts to establish clinically applicable predictive models.^122^

## 7 Conclusions

The gut microbiota acts as a dynamic interface that integrates host genetics, environmental exposures, and immune competence to shape vaccine efficacy. This review highlights the multifaceted role of the gut microbiota in fine-tuning vaccine-induced immunity through innate immune priming, metabolite-mediated immunomodulation, antigen-presenting cell reprogramming, and antigen cross-reactivity. The microbiota can either amplify or suppress vaccine responses, depending on their function and compositional equilibrium.

Heterogeneity in vaccine outcomes arises from the interplay between the microbiota and intrinsic host factors (such as age, sex, and immunosenescence) as well as extrinsic pressures (like antibiotic use or malnutrition). This positions the microbiota as a biogeographical mediator that translates environmental signals into distinct immunological phenotypes. While current interventions targeting the microbiota show promise, they encounter challenges related to scalability and variable effectiveness across different populations.

Emerging strategies, such as engineered probiotics that deliver TLR agonists and CRISPR-based microbiota editing, provide precision tools to address these challenges. Future research should focus on integrating multiomics approaches to clarify context-specific interactions between the microbiota and the immune system. Machine learning models that incorporate microbial diversity, metabolite profiles, and immune signatures could help predict vaccine responsiveness and inform personalized strategies.^80^

Ensuring equitable access to such technologies is critical for reducing global disparities, particularly in low- and middle-income countries, where dysbiosis and suboptimal vaccine performance often cooccur. By integrating microbial ecology with immunology and clinical practice, we can advance vaccination from empirical approaches to precision immunization, thereby optimizing protection across diverse populations.

Vaccine efficacy is shaped by bidirectional interactions between host-intrinsic factors (left) and extrinsic factors (right), mediated by the gut microbiota. Host-intrinsic factors include sex, age, genetic polymorphism, and pre-existing immunity. Extrinsic factors encompass antibiotic use, dietary habits, and geographic location. These factors collectively modulate microbiota composition and function, influencing vaccine efficacy. The image was created at BioRender.com.

(A) Innate immune sensing of microbial ligands. Flagellin and other microbe-associated molecular patterns are detected by Toll-like receptor 5 (TLR5) on innate cells via a MyD88-dependent cascade, providing adjuvant-like signals that enhance B-cell activation and antibody production. (B) Metabolite-mediated immunomodulation. Dietary fiber fermentation by commensals generates short-chain fatty acids (SCFAs), which cross the intestinal epithelium to upregulate genes involved in plasma cell differentiation and antibody class switching, thereby enhancing systemic IgG and mucosal secretory IgA (sIgA) responses. (C) Reprogramming of antigen-presenting cells. Gut microbiota-derived signals stimulate plasmacytoid dendritic cells (pDCs) to secrete type I interferon (IFN-α/β) and conventional DCs (cDCs) to produce proinflammatory cytokines (e.g., TNF-*α* and IL-6). These signals rewire DC metabolic and epigenetic programs, thereby enhancing the priming of naïve CD4⁺ T-cells into Th1, Th17, or T follicular helper (Tfh) lineages during antigen presentation. (D) Antigen cross-reactivity and immune memory. Microbial epitopes that structurally mimic vaccine antigens drive the expansion of cross-reactive T and B-cell clones before vaccination. These pre-existing memory populations may accelerate or bias subsequent vaccine-specific responses. *Image was Created at BioRender.com.*
